# How beliefs get in the way of the acceptance of evolutionary psychology

**DOI:** 10.3389/fpsyg.2014.01212

**Published:** 2014-11-11

**Authors:** Peter K. Jonason, Laura K. Dane

**Affiliations:** ^1^School of Social Sciences and Psychology, University of Western SydneyMilperra, NSW, Australia; ^2^Psychology, Douglas CollegeNew Westminster, BC, Canada

**Keywords:** evolutionary psychology, biases, belief systems, social psychology, philosophy

There can be no doubt there is serious resistance to evolutionary psychology (EP) as a theoretical paradigm from both within the field (e.g., social psychology) and in other disciplines (e.g., social sciences). Numerous researchers (Harris, 2003; Eastwick et al., 2014) appear to have made it their objective to show how predictions made and studies conducted by evolutionary psychologists are flawed (and even outright sexist). Such research programs have left evolutionary psychologists scratching their heads with the simple, yet fundamental question of why is everyone not an evolutionary psychologist?

Darren Burke (DB) details institutional biases in promoting evolutionary sciences. In the United States this shows itself in the debate about teaching Creationism or Intelligent Design in schools as an apparently reasonable effort to be balanced. Ironically, evolved mechanisms for extended collaborative actions with kin and non-kin to exploit resources may be responsible for financial, political, and intellectual collusions to maintain the very belief systems that spawned them. These may create institutional blocks in terms of funding, publishing, and hiring practices. As academics trained in psychology as well as biology and anthropology, we focus on individual-level obstacles to complement DB's position. We focus on the potential psychological reasons behind the resistance to EP.

We contend there are essentially four different types of psychological resistance to EP, all of which are a function of an individual's philosophical belief systems, whether they are implicit or explicit. The biases are not unique to mainstream psychology or even researchers but, instead, may be endemic in people, more generally. We focus our attention on researchers because we wish to draw attention to biases in those who have been educated to be less biased. This is not to say we are not biased in our own way. We, like most evolutionary psychologists, assume human beings are part of the natural world; the only explanations worth attending to are naturalistic; and the brain (and all that comes from it) is a naturally occurring, evolved aspect of humans. Assumptions pervade all of science, what matters is holding the fewest and most reasonable assumptions possible. We feel the theory of evolution offers just that, but there may be a series of psychological blocks that exclude individuals from thinking clearly about evolution in reference to humans and to eschew what evolutionary predictions/findings mean.

## Religious thinking

The first, most obvious objection stems from the denial of evolution *en toto*. Such beliefs normally stem from religious beliefs about the nature of the universe, human's place it in, and the active effort to maintain those beliefs. Many liberal and famous academics (e.g., Stephen Jay Gould) walk the line between belief in the supernatural/metaphysical and science by arguing that science and religion deal with “non-overlapping magisteria” (Gould, 1998, p. 274). This political view is functional for many scientists who, for want of protecting their own beliefs (often implicitly), protecting their own reputation, ensuring they do not lose their jobs, and securing funding from government agencies who are likely staffed by people who fundamentally disagree with all things related to evolution, may steer away from such topics or paradigms. However, psychological science (in particular) does overlap with questions of moral value and reasoning, and some religious claims overlap with scientific empiricism. Objections to the evolutionary study of human behavior, from this perspective, are hard to overcome because the motivation behind the denial stems from a (perceived) need to protect one's values and morals. If science is a pursuit of empirical evidence to support or falsify predictions derived from theory there can be no doubt that naturalistic predictions, derived from evolutionary theory are not only sound but must have relevance to human beings.

## Human exceptionalism

The second philosophical objection centers around a Spencerian version of the Darwin-Wallace theory of evolution. Many psychological and social scientists accept the theory of evolution in principle but deny that it is relevant to studying human cognition or behavior (i.e., Cartesian Dualism). Many can accept that our bipedal gate, relative hairlessness, or cranial structures have evolved through natural/sexual selection, but an application of the same principles to human behavior receives a vehement rejection. This position has implicitly haunted psychology for decades and we can see it today in the constant attempts to define what makes humans special relative to other animals (e.g., language; Pinker, 1994; culture; Henrich and McElreath, 2003; play; Maestripieri, 2012) and the implicit belief that the human brain needs its own science that is separate from biology. While these objections do not necessarily come from a religious background, there is an underlying sense that a naturalistic approach to human behavior threatens existing views of morals or ethics (see Curry, 2006).

## Environmental determinism

The third philosophical objection comes from those who may allow biology some role in explaining human behavior but the role is extremely limited. The obvious one of relevance comes in many names: The Standard Social Sciences Model, Tabula Rasa, and Environmental Determinism. All of these hold at their core the personal, political, or professional “need” to believe that human beings are more a product of environmental influences than evolved differences (see Genetic Determinism below). This ideology was most strongly expressed in behaviorism but still is predominant in social psychology textbooks and conferences. For instance, Worchel and Cooper (1976) say that “social psychology is the study of the way in which individuals are affected by social situations” (p. 7) and Shaver (1977) says social psychology is “the scientific study of the personal and situational factors that affect individual social behavior” (p. 14). This bias in focusing on the environmental, social, or cultural causes of human behavior is functional in that it allows researchers to suggest ways we can change behavior. This position is not all bad, except it leads to a pure focus on proximate mechanisms. This is not to say that all behaviors have ultimate, evolutionary causes, but an understanding of the potential ultimate functions of various aspects of human nature can lead to an even better understanding of how to change behavior. Nevertheless, in both cases researchers need to be more critical about understanding their topic of interest in macroscopic and microscopic levels (Bingham and Souza, 2009).

## Genetic determinism

Not only is there a gross misunderstanding of the theory of evolution (and its application to human behavior), but also there seems to be an active bias against learning about genetics and comparative biology. These “inferential prisons” leave researchers hard-pressed to explain many observed effects (e.g., twin concordance in personality; Vernon et al., 2008) and they are at a disadvantage compared to evolutionary psychologists whose models are expressly about the interaction of the person and the environment. EP and related disciplines like evolutionary developmental psychology (Evo Devo) ARE environmentalist disciplines. Take as an example the research on kin recognition and incest avoidance (Lieberman et al., 2007). The authors propose an innate learning process which, helps us determine who our siblings are (and therefore who to help and who not to mate with) through the length of sibling co-residence and the other child's perinatal association with one's mother. The degree to which these “environmental” factors are present depends on whether the individual is an older or younger sibling. A second born will of course not be exposed to cues of her sibling's perinatal association with their mother but will have cues to co-residence if she is raised with her sibling. This research is consistent with the Westermark hypothesis (1981), which fell out of favor in the 20th century partially because the SSSM belief that behavior is predominately environmentally determined. But if an evolutionary approach is consistent with environmental determinants of human behavior then why do many psychologists have issues with the discipline? The difference is that many social psychologists hold an implicit belief in a version of tabula rasa, or general purpose learning in humans (Lieberman and Symons, 1998). The Westermark hypothesis, like discussion of kin recognition mechanisms, leads many to think of genetically determined, automatic systems that tell us exactly who we are related to. This could not be farther from what the comparative biologists and evolutionary psychologists are arguing. Our brains estimate relatedness based on cues (environmental contingencies) that must be experienced through development (learning). The end result is not perfect knowledge of who we are related to, but best guesses based on available information that, on average over time, would have lead us to make decisions that increased inclusive fitness. We would argue that these specific cues and their specific effects on sibling altruism and incest aversion could only have been predicted from a perspective taking the evolved function of kin recognition into account.

DB argues for increased training in evolutionary theory, which logically should temper many of the objections to evolutionary approaches to psychology. However, we suggest that attention should be paid to the underlying motivations behind the critiques from social scientists. As long as EP is perceived to threaten political (e.g., men and women should be equal), moral (e.g., humans should be inherently nice), professional (e.g., all behavior is changeable), and religious (e.g., God created us in our present, immutable form) belief systems, the cognitive biases underlying those systems will act to preserve them. We fear that without the anchor that is Evolutionary Theory (see Confer et al., 2010), psychology as a science will continue to pitch and yaw through the sea.

### Conflict of interest statement

The authors declare that the research was conducted in the absence of any commercial or financial relationships that could be construed as a potential conflict of interest.
